# Immunohistochemical Study of the Tumor Immune Microenvironment in p16-Positive and p16-Negative Oral Squamous Cell Carcinoma and Its Prognostic Implications

**DOI:** 10.3390/diagnostics16091283

**Published:** 2026-04-24

**Authors:** Ingrid-Denisa Barcan, Tudor-Stelian Stoia-Djeska, Marina Rakitovan, Flavia Zara, Raluca Maria Closca, Alexandru Cristian Cindrea, Andreea-Mihaela Banta, Anda Gabriela Militaru, Horatiu Urechescu, Ioana Delia Horhat

**Affiliations:** 1 ENT Department, University of Medicine and Pharmacy “Victor Babes”, 300041 Timisoara, Romania; 2 ENT Department, Emergency City Hospital, 300254 Timisoara, Romania; 3 Department of Microscopic Morphology, University of Medicine and Pharmacy “Victor Babes”, 300041 Timisoara, Romania; 4 Angiogenesis Research Center, University of Medicine and Pharmacy “Victor Babes”, 300041 Timisoara, Romania; 5 Oro-Maxillo-Facial Surgery Clinic, Emergency City Hospital, 300062 Timisoara, Romania; 6 Department of Pathology, Emergency City Hospital, 300254 Timisoara, Romania; 7 Doctoral School, Faculty of General Medicine, University of Medicine and Pharmacy “Victor Babes”, 300041 Timisoara, Romania; 8 Emergency Department, Emergency Clinical Municipal Hospital, 300254 Timisoara, Romania; 9 Department of Internal Medicine I, Medical Semiology I, University of Medicine and Pharmacy “Victor Babes”, 300041 Timisoara, Romania; 10 Department of Oral and Maxillo-Facial Surgery, Faculty of Dental Medicine, University of Medicine and Pharmacy “Victor Babes”, 300041 Timisoara, Romania

**Keywords:** oral squamous cell carcinoma, tumor immune microenvironment, immunohistochemistry

## Abstract

**Background/Objectives:** Oral squamous cell carcinoma (OSCC) is a tumor characterized by heterogeneous clinical behavior and prognosis. The tumor immune microenvironment plays a significant role in tumor progression and patient prognosis. p16 expression has been investigated as a surrogate biomarker in certain subtypes of head and neck squamous cell carcinomas, but its prognostic significance in oral squamous cell carcinoma remains incompletely elucidated. **Methods:** A retrospective cohort of 59 patients diagnosed with primary oral squamous cell carcinoma was analyzed. Tumor samples were evaluated for p16 expression and immunohistochemical markers associated with immune cell populations. Associations between immune microenvironment features, p16 status, and clinical outcomes such as recurrence and survival rate were analyzed. **Results:** p16-positive tumors were predominantly associated with immunotype A and exhibited higher densities of CD8+ cytotoxic T lymphocytes and natural killer (NK) cells. In contrast, immunotype B tumors showed similar characteristics regardless of p16 status, with no significant differences between p16-positive and p16-negative cases. Distinct immune profiles were variably associated with clinicopathological features and patient outcomes. **Conclusions:** These findings suggest that the immunological phenotype of oral squamous cell carcinoma may represent a potential prognostic factor.

## 1. Introduction

Oral squamous cell carcinoma accounts for approximately 90% of oral malignancies. Despite advances in diagnostic and therapeutic strategies, OSCC continues to be associated with significant morbidity and mortality, and the five-year survival rate has shown only limited improvement over the past decades [1,2].

The tumor microenvironment (TME) has emerged as an important factor in cancer progression and response to therapy. The TME represents a complex system in which tumor cells interact with stromal and immune components, influencing tumor growth, invasion, angiogenesis, metastasis, and immune evasion [3,4].

A particularly important component of the TME is the tumor immune microenvironment (TIME), which includes different subsets of immune cells such as T lymphocytes (CD3, CD4, CD8) and B lymphocytes (CD20). Additional immune cell populations present in the TME include antigen-presenting cells (CD1A), macrophages (CD68), and mast cells (CD117), which may also contribute to tumor progression and immune regulation [3,4,5]. The density, distribution, and functional status of these immune cell populations may play a crucial role in determining tumor behavior and patient prognosis. Several studies have demonstrated that a high infiltration of cytotoxic CD8+ T lymphocytes is generally associated with a more effective antitumor immune response and improved survival outcomes, while certain immunosuppressive mechanisms within the microenvironment may promote tumor progression and resistance to therapy [6,7,8].

The etiopathogenesis of OSCC is multifactorial. Traditional risk factors include tobacco use and alcohol consumption; however, infection with high-risk human papillomavirus (HPV), particularly HPV-16, is also involved in a subset of cases [9,10]. Overexpression of p16 is widely used as a surrogate immunohistochemical marker for HPV-associated carcinogenesis and is considered to have important biological and prognostic implications [9]. HPV-related tumors may present distinct molecular and clinical characteristics compared with HPV-negative cases, which makes evaluating p16 expression relevant in studies investigating tumor biology and patient outcome [2,10].

Given the growing evidence regarding the impact of the tumor immune microenvironment on tumor progression and patient outcome, a detailed characterization of the cellular components of the tumor immune microenvironment in OSCC may provide valuable insights into tumor biology [5,11,12]. Furthermore, the immunohistochemical evaluation of these immune cell populations, as well as their correlation with clinicopathological parameters and patient survival, may contribute to a better understanding of the immune microenvironment and its role in OSCC progression and prognosis [5,6].

Although there are currently numerous studies investigating the role of the tumor immune microenvironment in head and neck cancers, data specifically focusing on oral squamous cell carcinoma remain limited and often heterogeneous. Most studies assess individual immune cell populations or rely on distinct methodologies, making comparisons difficult and limiting the development of reproducible assessment strategies. Furthermore, the integration of multiple immune cell subsets into a unified semiquantitative framework, along with their correlation with clinicopathological parameters and patient outcomes, has not been sufficiently explored in oral squamous cell carcinoma. While previous studies have described the prognostic significance of individual tumor-infiltrating immune cell populations in OSCC, the present study focuses on the integration of these components into distinct immune patterns. The novelty of this work does not lie in the assessment of individual markers per se, but rather in the identification of immune pattern-based subtypes, which offer a more comprehensive and biologically coherent characterization of the tumor immune microenvironment and its associated prognostic heterogeneity.

In addition, although p16 overexpression is widely accepted as a surrogate marker of HPV-driven carcinogenesis in oropharyngeal squamous cell carcinoma, its significance in oral squamous cell carcinoma is less studied and remains a subject of ongoing debate. The relationship between p16 expression and the tumor immune microenvironment in oral cancer is not fully understood, and further investigations may help clarify its biological relevance and potential prognostic significance in this specific tumor type.

Therefore, the aim of the present study was to characterize and quantify the cellular components of the tumor immune microenvironment in oral squamous cell carcinoma using immunohistochemical methods, to integrate these findings into a semiquantitative scoring system, and to evaluate their correlations with clinicopathological parameters, p16 expression, and patient prognosis.

## 2. Materials and Methods

### 2.1. Patient Selection and Inclusion Criteria

**Figure 1:**
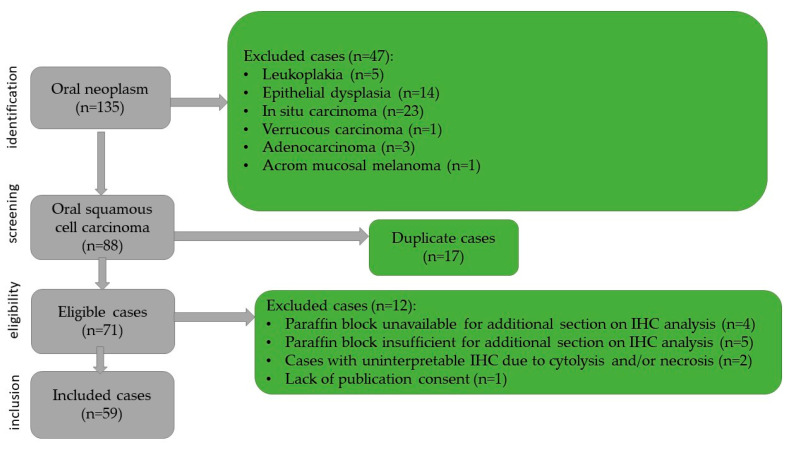
Flowchart of the case selection.

This retrospective study included 59 patients admitted to the Otorhinolaryngology and Maxillofacial Surgery Departments of the Emergency Hospital of Timișoara between January 2019 and December 2020, diagnosed with oral squamous cell carcinoma. Case identification was based on the specimen reception registers of the Pathology Department, while clinical data were retrieved from the hospital’s electronic medical records. For each patient, the following clinico-epidemiological parameters were collected: age, sex, tobacco/alcohol use, and the size and location of the tumor. Histopathological parameters such as the histological subtype of the tumor, degree of keratinization, histological grading, depth of invasion, worst pattern of invasion, presence of ulceration, lymphatic and/or vascular invasion, tumor stage, and resection margin status were noted. Oncological treatment and patient outcome were also noted. Survival data were obtained from the Central Population Registry for the period between January 2019 and December 2025. The inclusion criteria comprised patients aged over 18 years, presence of a primary oral tumor, histopathological confirmation of squamous cell carcinoma, complete clinical and imaging data, complete surgical excision with free margins, and the availability of adequate paraffin-embedded tissue blocks for immunohistochemical analysis. The exclusion criteria from the study were the following: leukoplakia, dysplasia and carcinoma in situ lesions, verrucous carcinomas and adenocarcinomas, other tumor cell lines (including achromic melanoma), as well as cases with unavailable or insufficient paraffin blocks. Tumors with incomplete surgical excision and those with marked cytolysis, unsuitable for immunohistochemical interpretation, were also excluded from this study. The case selection process and exclusion criteria are illustrated in Figure 1.

### 2.2. Ethical Considerations

This study was conducted in accordance with Romanian legislation and the ethical principles outlined in the Declaration of Helsinki. Written informed consent was obtained from all patients, and the corresponding biopsy consent documentation was reviewed and verified. Histological slide preparation was performed in compliance with the recommendations of the Ministry of Health and established international guidelines. Ethical approval was obtained from the Scientific Research Ethics Committee of the Victor Babeș University of Medicine and Pharmacy, Timișoara, (Project identification code: 18) on 23 February 2026. Patient data were deidentified before conducting the statistical analysis.

### 2.3. Laboratory Technique

The harvested specimens were fixed in 4% buffered formaldehyde. Serial sections of 4 μm thickness were prepared using a semi-automated Leica RM2235 rotary microtome (Leica Biosystems, Nussloch, Germany) and mounted on SuperFrost™ glass slides (St. Louis, MO, USA). Routine hematoxylin and eosin staining was performed for morphological assessment. The following histopathological parameters were evaluated for each tumor: histological type, microscopic grading, degree of keratinization, inflammatory infiltrate, presence of perineural and/or angiolymphatic invasion, depth of invasion (DOI), worst pattern of invasion (WPOI-5), resection margin status, and the presence or absence of lymph node metastases.

Immunohistochemical analysis was performed to assess and quantify the tumor immune microenvironment using the following primary antibodies: anti-CD3, anti-CD4, anti-CD8, anti-CD20, anti-CD56, anti-CD68, anti-CD1a, and anti-CD117. The anti-p16 antibody has been used as a marker for HPV infection. All immunohistochemical procedures were conducted using the Leica Bond-Max automated staining system (Leica Biosystems Melbourne Pty Ltd., Waverley, Australia) in accordance with the manufacturer’s standardized protocols. All antibodies and reagents used for immunohistochemical staining were sourced from Leica Biosystems (Newcastle, UK). Detailed information regarding the immunohistochemical reagents, including the antibody type, clone, and dilution, is presented in Table 1. Immunohistochemical staining was performed on serial sections of 3 μm thickness, with each tumor section incubated with a single primary antibody.

The slides were independently evaluated by two pathologists, each assessing 10 high-power fields at 40× magnification per section. Prior to study, a calibration session was performed on a subset of cases to harmonize the scoring criteria. Interobserver discrepancies were resolved by joint review and consensus.

Histopathological grading was performed based on the nucleus-to-cytoplasm ratio, nuclear pleomorphism, mitotic index, degree of atypia, and areas of keratinization. Thus, the carcinoma tumors were classified as G1 (well differentiated), G2 (moderately differentiated, and G3 (poorly differentiated). Tumor staging (pTMN stage) and reporting of depth of invasion (DOI) and worst pattern of invasion (WPOI-5) were performed according to the TNM classification system and the recommendations of the 8th edition of the American Joint Committee on Cancer.

The tumor immune microenvironment was evaluated with a semi-quantitative scoring system developed by the authors, using the density of positively stained immune cells. For each case, positive immune cells were counted in 10 representative high-power fields at 40× magnification, and the average number of cells was used for scoring. The scoring system was defined as follows: score 0, ≤9 positive immune cells; score 1, 10–49 positive immune cells; score 2, 50–99 positive immune cells; and score 3, >100 positive immune cells per 10 fields at 40× objective. This approach was designed to provide a reproducible estimation of immune cell infiltration based on previously reported semi-quantitative methods used in histopathological studies. According to the resulting score, tumors were classified into two immune patterns: type A (immune-hot tumor) and type B (immune-cold tumor). For p16 expression, immunopositivity was defined as strong nuclear and/or cytoplasmic staining in more than 70% of tumor cells.

### 2.4. Objectives

The objectives of this study were primarily to identify and quantify the cellular elements of the tumor immune microenvironment of oral squamous cell carcinoma. Subsequently, potential statistical correlations between immune components of the tumor microenvironment and histopathological parameters with prognostic or predictive value were evaluated.

### 2.5. Statistical Analysis

Data analysis and graphical representation were performed using R version 4.5.1 (R Foundation for Statistical Computing, Vienna, Austria). The following packages were used: dplyr, stringr, lubridate, janitor, readr, forcats, gtsummary, survival, survminer, ggpubr.

Continuous variables were summarized as the median and interquartile range (IQR), whereas categorical variables were reported as absolute counts and percentages. Age was compared between groups using the Wilcoxon rank-sum test. Categorical variables were compared between immunotype groups using Pearson’s chi-square test or the Fisher’s exact test, as appropriate.

The tumor immune microenvironment had been classified histologically into two immune patterns, type A (immune-hot) and type B (immune-cold), based on the semiquantitative immunohistochemical scoring system described above, while p16 positivity was defined as strong nuclear and/or cytoplasmic staining in more than 70% of tumor cells. Statistical analyses were focused on the association of the immunotype with clinicopathologic variables, p16 status, recurrence, and survival outcomes.

Overall survival (OS) was defined as the interval from the date of diagnosis to the date of death from any cause or censoring as of 31 December 2025. Recurrence-free survival (RFS) was defined as the interval from diagnosis to the first documented recurrence. Patients without recurrence were censored at the date of death or, if alive, at the study end date. Kaplan-Meier curves were generated for the whole cohort and stratified by immunotype, sex, smoking status, histologic grade, p16 status, depth of invasion, chemotherapy, and radiotherapy. Survival differences between groups were assessed using the log-rank test.

Univariable Cox analysis was first performed for immunotype, followed by small adjusted models centered on immunotype, including age or p16 status as covariates. Because the number of death events was limited, multivariable survival models were intentionally restricted to a small number of predictors. DOI was explored descriptively, but adjusted Cox modeling with DOI was interpreted cautiously because of the complete separation of events across DOI strata. Results of the Cox models were reported as hazard ratios (HRs) with 95% confidence intervals (CIs). The proportional hazards assumption was assessed using Schoenfeld residuals.

Landmark overall survival estimates were calculated at 6, 12, 36, and 48 months for the whole cohort and at 12, 36, and 48 months according to immunotype.

All statistical tests were two-tailed, and a *p* value < 0.05 was considered statistically significant.

## 3. Results

### 3.1. Clinical and Demographic Data

The study cohort consisted of 59 patients, including 45 males (76%) and 14 females (24%), with ages at diagnosis ranging from 31 to 84 years and a median age of 62 years. A history of smoking was reported in 46 patients (78%).

The most common tumor sites were the dorsal surface of the tongue (13, 22%) and the lower labial mucosa (11, 19%), followed by the anterior floor of the mouth (10, 17%) and the lateral border of the tongue (9, 15%). Less frequent sites included the ventral surface of the tongue (6, 10%), the palatoglossal arch (3, 5.1%), the upper labial mucosa (2, 3.4%), and the oral commissure, mandibular alveolar ridge, anterior two-thirds of the tongue, buccal mucosa, and hard palate (1 each, 1.7%).

Additional clinical and epidemiological characteristics, together with treatment and outcome data, are presented in Table 2.

At the time of data collection, 15 patients (25.4%) were deceased.

### 3.2. Morphological Features

Microscopic examination on hematoxylin-eosin staining showed that conventional squamous cell carcinoma was the predominant histologic subtype, accounting for 40 cases (68%). Basaloid squamous cell carcinoma was identified in nine patients (15%), while acantholytic, papillary, and spindle cell variants were less frequent, being observed in five (8.5%), three (5.1%), and two cases (3.4%), respectively.

Histologic grading revealed a predominance of moderately differentiated tumors (G2, 45, 76%), followed by poorly differentiated tumors (G3, 8, 14%) and well-differentiated tumors (G1, 6, 10%).

Pathologic T stage was most frequently represented by T2 tumors (25, 42%), followed by T1 (22, 37%), T3 (11, 19%), and T4a (1, 1.7%). Regarding nodal status, most tumors were classified as N0 (28, 47%), whereas the N1, N2a, N2b, N2c, and N3b categories were less common. Perineural invasion was present in 24 cases (41%), while lymphovascular invasion was observed in 20 cases (34%).

The depth of invasion was 5–10 mm in most tumors (30, 51%), followed by <5 mm (19, 32%) and >10 mm (10, 17%). The worst pattern of invasion was present in 22 cases (37%) (Table 3).

### 3.3. Immunohistochemical Features

#### 3.3.1. Immunohistochemical Features of p16-Negative Tumors

The majority of oral squamous carcinomas (*n* = 45, 76.3%) were p16 negative tumors, with moderate to weak intensity reaction in less than 70% of tumor cells.

**Figure 2:**
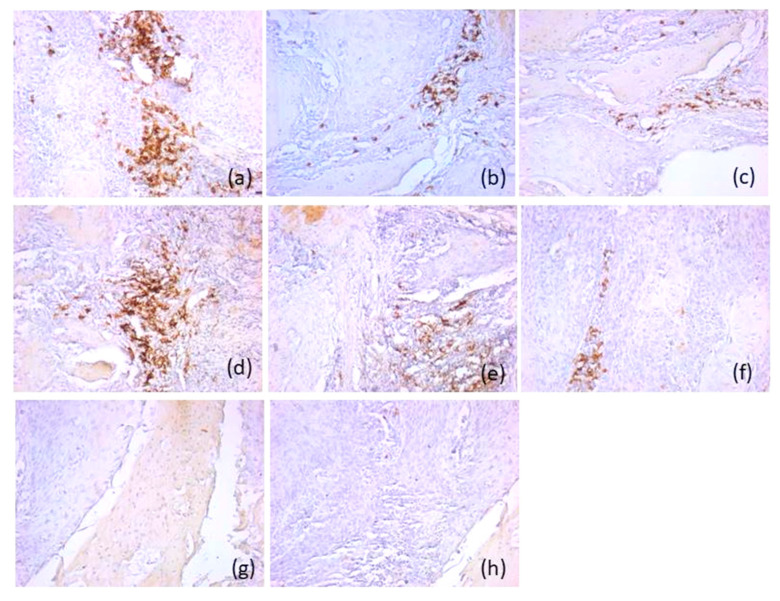
Representative immunohistochemical profile of p16-positive tumors of immunotype A, ob.20×: (**a**) CD20 antibody; (**b**) CD3 antibody; (**c**) CD4 antibody; (**d**) CD8 antibody; (**e**) CD56 antibody; (**f**) CD1a antibody; (**g**) CD117 antibody; (**h**) CD68 antibody. Images are representative of the dominant immune pattern.

Based on their immunoprofile, we classified p16 negative tumors into two categories. Therefore, most p16-negative carcinomas (*n* = 32, 71.1%) were type A immune-hot tumors, with an active immunotype. The tumors had an increased density of CD20-positive B lymphocytes, and CD8-positive T lymphocytes, with an immunoscore of 2; a moderate density of CD3- and CD4-positive T lymphocytes, and CD1a-positive antigen-presenting dendritic cells, with an immunoscore of 1. The density of CD56-positive natural killer lymphocytes was lower compared to CD8-positive T lymphocytes. It was also found that they presented a lower distribution in the tumor component (score 1) compared to the adjacent normal mucosa (score 1 and 2). These tumors were associated with rare or absent CD117-positive mast cells and CD68-positive macrophages (score 0). T lymphocytes were distributed both peritumorally and intratumorally, while B lymphocytes were predominantly intratumoral. Antigen-presenting dendritic cells had an increased density in the peritumoral areas and in the adjacent mucosa (score 1) (Figure 2).

**Figure 3:**
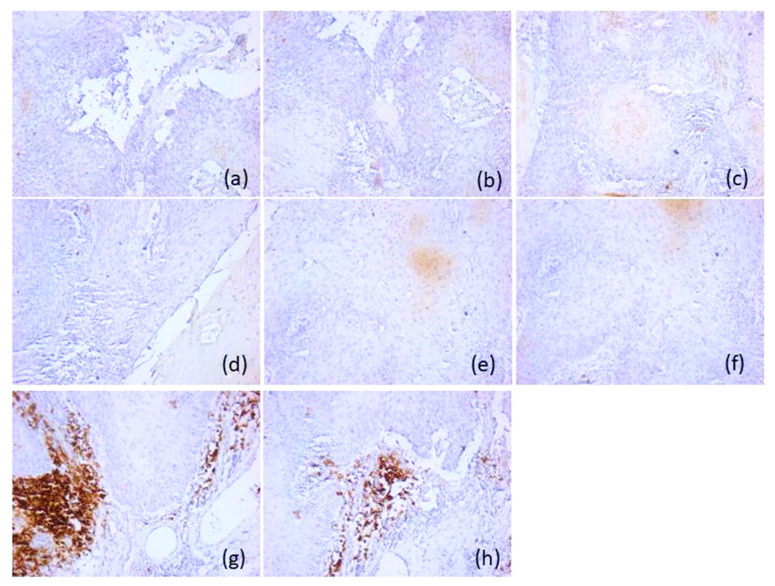
Representative immunohistochemical profile of p16-positive tumors of immunotype B, ob.20×: (**a**) CD20 antibody; (**b**) CD3 antibody; (**c**) CD4 antibody; (**d**) CD8 antibody; (**e**) CD56 antibody; (**f**) CD1a antibody; (**g**) CD117 antibody; (**h**) CD68 antibody. Images are representative of the dominant immune pattern.

The remaining 13 patients (28.9%) negative for p16 protein were type B, presenting a microenvironment with marked immunosuppression. These tumors were classified as immune-cold tumors, with depletion of CD20-positive lymphocytes, CD3-, CD4- and CD8-positive T lymphocytes and CD1a-positive antigen-presenting dendritic cells absent or rare (score 0), but an increased density of CD68-positive macrophages (score 2 and 3) and CD117-positive mast cells (scores 3), both in tumoral and stromal area. The density of CD56-positive natural killer lymphocytes was low compared to the active immune subtype, with scores of 0 (*n* = 11, 84.6%) and 1 (*n* = 2, 15.4%) being predominantly recorded (Figure 3).

#### 3.3.2. Immunohistochemical Features of p16-Positive Tumors

Only 23.7% of oral squamous cell carcinomas (*n* = 14) were p16-positive, with intense and diffuse reaction in 70–100% of the tumor cells, showing both nuclear and cytoplasmic immunostaining.

**Figure 4:**
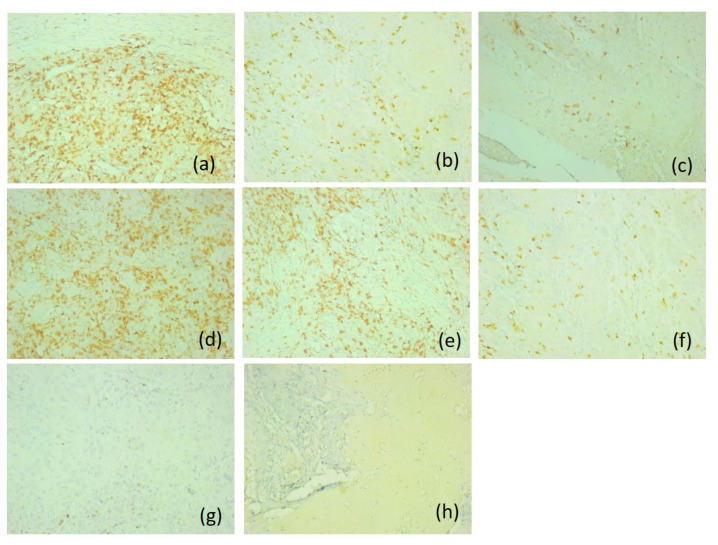
Representative immunohistochemical profile of p16-negative tumors of immunotype A, ob.20×: (**a**) CD20 antibody; (**b**) CD3 antibody; (**c**) CD4 antibody; (**d**) CD8 antibody; (**e**) CD56 antibody; (**f**) CD1a antibody; (**g**) CD117 antibody; (**h**) CD68 antibody. Images are representative of the dominant immune pattern.

The majority of these tumors were type A (*n* = 11, 78.6%), with activated immunotype hot, presenting higher scores for both CD20-positive B lymphocytes and CD8-positive T lymphocytes (score 3), compared to p16-negative tumors. A very high density of CD56-positive NK lymphocytes (score 3) was also observed, both in the tumoral and peritumoral areas and in the mucosa adjacent to the tumor. CD117-positive mast cells, CD68-positive macrophages and CD1a antigen-presenting dendritic cells presented similar scores to p16-negative tumors (score 0, score 1, respectively) (Figure 4).

**Figure 5:**
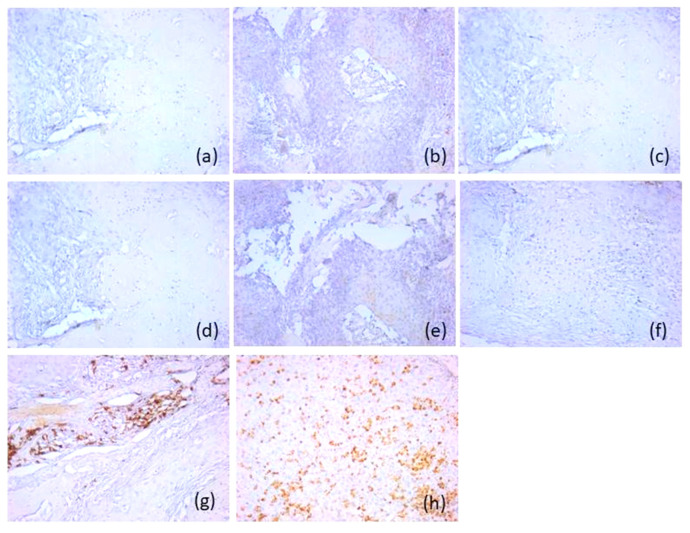
Representative immunohistochemical profile of p16-negative tumors of immunotype B, ob.20×: (**a**) CD20 antibody; (**b**) CD3 antibody; (**c**) CD4 antibody; (**d**) CD8 antibody; (**e**) CD56 antibody; (**f**) CD1a antibody; (**g**) CD117 antibody; (**h**) CD68 antibody. Images are representative of the dominant immune pattern.

Three p16-positive cases (21.4%) were classified as subtype B, with a suppressive immune microenvironment. These showed similar microscopic characteristics to the immune microenvironment of p16 negative immune-cold tumors (Figure 5).

### 3.4. Survival Analysis

In the overall cohort, 15 of 59 patients died during follow-up. The estimated overall survival rates were 100% at 6 months, 98.3% at 12 months, 86.4% at 36 months, and 84.7% at 48 months. Recurrent disease occurred in 13 patients, accounting for a total of 14 recurrence events, as one patient developed two separate recurrences.

The OS rates at 12, 36, 48 and 72 months were 58%, 51%, 50% and 45%, respectively.

**Figure 6:**
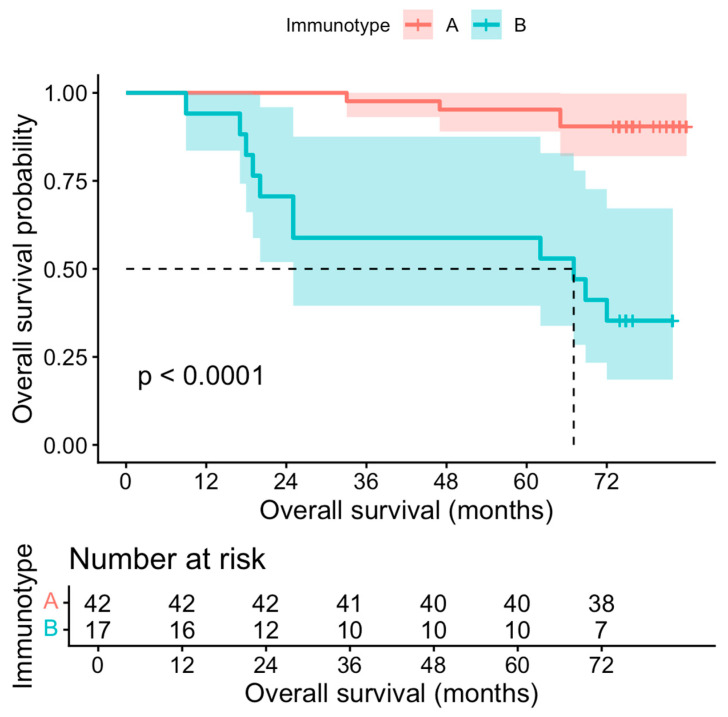
Kaplan-Meier OS and survival curves for patients by immunotype.

**Figure 7:**
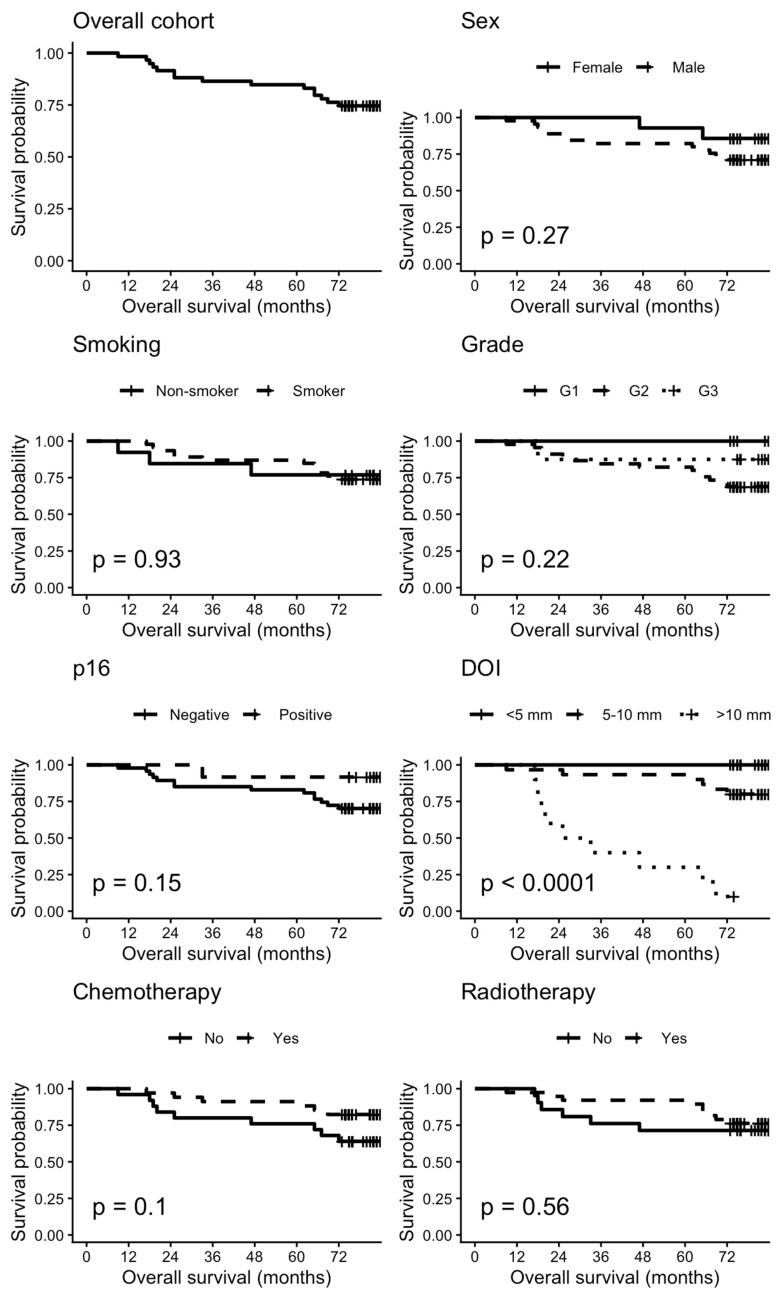
Kaplan-Meier survival curves.

The overall survival differed noticeably according to the immunotype. Among the 42 patients classified as immunotype A, 4 deaths were recorded, compared with 11 deaths among the 17 patients classified as immunotype B. Kaplan-Meier analysis (Figure 6) showed that the estimated 12-, 36-, and 48-month overall survival rates for immunotype A were 100%, 97.6% (95% CI 93.1–100.0), and 95.2% (95% CI 89.0–100.0), respectively. In contrast, the corresponding survival rates for immunotype B were 94.1% (95% CI 83.6–100.0), 58.8% (95% CI 39.5–87.6), and 58.8% (95% CI 39.5–87.6), respectively. This difference was statistically significant on log-rank analysis (chi-square = 24.1, *p* < 0.001). In the univariable Cox regression, immunotype B was associated with a significantly increased risk of death compared with immunotype A (HR 10.31, 95% CI 3.26–32.54, *p* < 0.001). These findings are highlighted in Figure 7. Immunotype B remained independently associated with worse overall survival after adjustment for age (adjusted HR 10.23, 95% CI 3.23–32.42, *p* < 0.001) and after adjustment for p16 status (adjusted HR 9.85, 95% CI 2.73–35.46, *p* < 0.001).

The recurrence-free survival also differed according to the immunotype. Recurrence occurred in 7 of 42 patients (16.7%) with immunotype A and in 6 of 17 patients (35.3%) with immunotype B. Kaplan-Meier analysis showed significantly shorter recurrence-free survival in the immunotype B group (log-rank chi-square = 4.88, *p* = 0.03).

Figure 7 depicts the Kaplan-Meier curves for the overall cohort and several subgroups.

## 4. Discussion

The tumor immune microenvironment plays a fundamental role in the development, progression, and clinical behavior of oral squamous cell carcinoma [13,14,15,16]. In recent years, increased attention has been directed towards the clinical and translational relevance of the tumor microenvironment in head and neck malignancies [17,18]. Increasing evidence suggests that interactions between tumor cells and immune components within the tumor microenvironment significantly influence tumor growth, invasion, immune evasion, and response to therapy [4,19]. The OSCC microenvironment represents a dynamic ecosystem composed of malignant epithelial cells, stromal components, extracellular matrix, and a wide variety of immune cells, including T lymphocytes, B lymphocytes, macrophages, dendritic cells, mast cells, and natural killer cells [4,16].

In the present study, we analyzed the immune landscape of OSCC using immunohistochemical markers representing key immune cell populations, while also evaluating their relationship with p16 expression. Our results revealed two distinct immune phenotypes: an immune-active subtype (immunotype A) characterized by abundant lymphocytic infiltration and an immune-suppressed subtype (immunotype B) characterized by reduced lymphocyte density and an increased presence of macrophages and mast cells. These findings support the concept of immune-“hot” and immune-“cold” tumors, which have been increasingly described in head and neck cancers and are considered an important determinant of tumor immunogenicity and therapeutic responsiveness [20,21,22].

The classification of tumors into “hot” and “cold” immune phenotypes has emerged as a widely accepted framework for describing the tumor immune microenvironment. Immune-hot tumors are characterized by a high density of tumor-infiltrating immune cells and an active antitumor immune response, whereas immune-cold tumors exhibit minimal immune infiltration and are often associated with immune evasion mechanisms [23,24]. This conceptual model has been extensively described in several solid tumors, including head and neck squamous cell carcinomas, with important prognostic and therapeutic implications, particularly in the context of response to immunotherapy. Similar immune-based classifications have also been reported in oral squamous cell carcinomas, where tumors are generally classified into inflamed (immuno-hot) and non-inflamed (immuno-cold) phenotypes based on the density and activity of tumor-infiltrating immune cells [25]. In the present study, we applied this framework to oral squamous cell carcinoma by operationalizing immune infiltration through a semiquantitative scoring system based on the density of positively stained immune cells. Although our classification into type A (immuno-hot) and type B (immuno-cold) tumors is not derived from a standardized scoring system, it reflects biologically relevant differences in the tumor immune microenvironment and is consistent with previously reported approaches that distinguish inflamed from non-inflamed tumors. This simplified classification may provide a practical and reproducible method for routine histopathological evaluation, especially in settings where advanced molecular profiling is not available.

One of the most relevant findings of our study was the predominance of cytotoxic CD8+ T lymphocytes in immune-active tumors. CD8+ T cells represent the main effector population of the adaptive antitumor immune response and can recognize and eliminate malignant cells through antigen-specific cytotoxic mechanisms [26,27]. Previous studies have shown that increased densities of CD8+ tumor-infiltrating lymphocytes are associated with improved survival and better clinical outcomes in patients with OSCC and other head and neck squamous cell carcinomas [21,28]. The presence of these cells within the tumor microenvironment suggests the existence of active immune surveillance mechanisms that may contribute to limiting tumor progression [26].

In addition to cytotoxic T lymphocytes, our study demonstrated a considerable presence of B lymphocytes (CD20+) in tumors classified as immune-active. Although less investigated than T cells, B cells have recently been recognized as important regulators of tumor immunity. They may participate in antitumor responses through antibody production, antigen presentation, and modulation of T-cell activation. Furthermore, the presence of intratumoral B cells may be associated with the formation of tertiary lymphoid structures, which have been correlated with improved prognosis in several malignancies, including oral squamous cell carcinoma [21,29,30].

Dendritic cells expressing CD1A represent a key component of the antigen-presenting cell compartment, playing a crucial role in the initiation of adaptive immune responses. Their reduced density has been reported in OSCC, particularly in immunosuppressed tumors, suggesting a potential impairment of antitumor immunity [31].

Natural killer cells, identified in our study by CD56 expression, were particularly abundant in p16 positive tumors with an immune-active phenotype. NK cells represent an essential component of natural antitumor immunity [4,32,33,34] and can recognize transformed cells independently of antigen presentation through a balance of activating and inhibitory receptors. Previous studies have suggested that increased NK cell infiltration reflects enhanced immune recognition of tumor cells and may contribute to improved tumor control [4,32].

Tumors classified as immunotype B showed a markedly different immune profile characterized by reduced lymphocytic infiltration and increased densities of macrophages and mast cells. Tumor-associated macrophages (TAMs), identified by CD68 expression, represent one of the most abundant immune cell populations in the OSCC microenvironment and have been implicated in tumor progression through multiple mechanisms, including angiogenesis, extracellular matrix remodeling, immune suppression, and facilitation of tumor invasion and metastasis [35,36,37]. In many cancers, TAMs acquire an M2-like phenotype that promotes tumor growth and suppresses effective antitumor immune response [36].

Similarly, mast cells (CD117+) have been reported to contribute to tumor progression through the release of pro-angiogenic mediators, cytokines, and proteolytic enzymes that can promote tumor invasion and vascularization [38]. The increased density of macrophages and mast cells observed in immune-cold tumors in our cohort suggests the presence of a tumor-promoting microenvironment, which may facilitate immune evasion and tumor progression.

Another important aspect of our study was the evaluation of the relationship between p16 expression and the tumor immune microenvironment. P16 overexpression is widely used as a surrogate marker for HPV-associated carcinogenesis in OSCC [17]. In our cohort, p16-positive tumors represented approximately one quarter of cases and were predominantly associated with the immune-active phenotype. These tumors demonstrated higher densities of CD8+ T lymphocytes, B lymphocytes, and NK cells compared with p16-negative tumors.

The identification of immune phenotypes has important clinical implications. Immune-hot tumors characterized by abundant cytotoxic lymphocyte infiltration are generally considered more responsive to immunotherapeutic strategies, including immune checkpoint inhibitors [39,40]. In contrast, immune-cold tumors often demonstrate mechanisms of immune exclusion or immunosuppression. These mechanisms may also involve the contribution of cancer-associated fibroblasts, as suggested by recent studies [41,42]. Therefore, the characterization of the tumor immune microenvironment may contribute to improved patient stratification and the development of personalized therapeutic strategies. Additionally, previous clinical studies have contributed to the understanding of pathological processes in the head and neck region, supporting the complex biological context of OSCC [43,44].

Overall, our results support the growing body of evidence suggesting that the tumor immune microenvironment represents an important biological factor in OSCC progression [19]. The identification of distinct immune phenotypes and their relationship with p16 expression may provide valuable insight into tumor biology and may contribute to improved prognostic stratification and therapeutic decision-making in patients with OSCC.

This study has some limitations that should be acknowledged. The relatively small sample size may limit the statistical power of the analyses and the robustness of subgroup comparisons. Secondly, the inclusion criteria restricted the study population to patients who underwent complete surgical resection with histologically negative margins, which may introduce selection bias and limit the representativeness of the cohort. As a result, the findings may not be fully generalizable to patients with advanced disease, incomplete tumor resection, or those treated with non-surgical methods. Additionally, the retrospective design of the study may be associated with inherent limitations, including potential variability in clinical data collection.

Furthermore, HPV status was assessed only indirectly through p16 immunohistochemistry, without confirmatory HPV DNA testing. Although p16 is used as a surrogate marker in HPV-associated head and neck carcinomas, its reliability in oral squamous cell carcinoma is less well established, and a discordance between p16 expression and true HPV-driven disease has been reported. The lack of HPV DNA analysis, therefore, represents an additional limitation, as it may affect the accuracy of HPV-related interpretation and its potential correlation with the tumor immune microenvironment. Thus, the results should be interpreted with caution, and further validation in larger and more heterogeneous cohorts with comprehensive HPV DNA testing is warranted.

Finally, the relatively low number of death events may limit the statistical robustness of the survival analyses. In particular, this may affect the stability of hazard ratio estimates in the Cox regression models and reduce the overall power to detect significant associations. Therefore, the survival-related findings should be interpreted with caution and validated in larger and prospective lots.

## 5. Conclusions

This study provides an exploratory characterization of the tumor immune microenvironment in oral squamous cell carcinoma and suggests the presence of distinct immunophenotypes associated with p16 expression. Tumors with p16 positivity showed higher densities of CD8+ T lymphocytes and NK cells, indicating a more inflamed immune profile (immunotype A), whereas immunotype B tumors appeared largely independent of p16 status. These findings suggest that immune pattern-based classification may provide complementary information regarding the biological behavior of OSCC. Differences in overall survival and recurrence-free survival according to immunotype were observed; however, given the limited sample size and the small number of events, these results should be interpreted with caution.

Additional studies involving larger patient cohorts and longer monitoring are required to further clarify the prognostic significance of the tumor immune microenvironment and its relationship with p16 status in oral squamous cell carcinoma.

## Figures and Tables

**Table 1:** Antibodies used for immunohistochemical reactions.

Antibody	Substrate	Dilution	Clone	Cell Type
^1^ p16	Monoclonal, mouse	1:100	CS1	Malignant squamous cells
^2^ CD3	Monoclonal, mouse	1:500	LN10	T lymphocyte
^3^ CD4	Monoclonal, mouse	1:100	4B12	T helper lymphocyte
^4^ CD8	Monoclonal, mouse	1:500	4B11	T cytotoxic lymphocyte
^5^ CD20	Monoclonal, mouse	^6^ RTU	L26	B Lymphocyte
^7^ CD117	Monoclonal, rabbit	1:200	EP10	Mast cell
^8^ CD1a	Monoclonal, mouse	RTU	MTB1	Dendritic cell
^9^ CD68	Monoclonal, mouse	1:100	514H12	Macrophage
^10^ CD56	Monoclonal, rabbit	RTU	MRQ-42	Natural killer lymphocyte

^1^ p16 (inhibitor of cyclin-dependent kinase 4a); ^2^ CD3 (cluster of differentiation 3); ^3^ CD4 (cluster of differentiation 4); ^4^ CD8 (cluster of differentiation 8); ^5^ CD20 (cluster of differentiation 20); ^6^ RTU (ready-to-use); ^7^ CD117 (cluster of differentiation 117); ^8^ CD1a (cluster of differentiation 1a); ^9^ CD68 (cluster of differentiation 68); ^10^ CD56 (cluster of differentiation 56).

**Table 2:** Descriptive statistics of the entire cohort stratified by immunotype.

Variable	All Patients (*N* = 59)	Immunotype A (*N* = 42)	Immunotype B (*N* = 17)	*p*-Value
Age ^#^	62 (55.5–69.5)	62 (56–68.5)	61 (54–73)	0.602
Sex (male)	45 (76%)	31 (74%)	14 (82%)	0.753
Smoker	46 (78%)	31 (74%)	14 (82%)	0.921
Localization				0.055
Oral commissure	1 (1.7%)	–	1 (5.9%)	
Mandibular alveolar ridge	1 (1.7%)	–	1 (5.9%)	
Anterior two-thirds of the tongue	1 (1.7%)	–	1 (5.9%)	
Dorsal surface of the tongue	13 (22%)	10 (24%)	3 (18%)	
Ventral surface of the tongue	6 (10%)	3 (7.1%)	3 (18%)	
Buccal mucosa	1 (1.7%)	–	1 (5.9%)	
Lower labial mucosa	11 (19%)	8 (19%)	3 (18%)	
Upper labial mucosa	2 (3.4%)	1 (2.4%)	1 (5.9%)	
Lateral border of the tongue	9 (15%)	9 (21%)	–	
Hard palate	1 (1.7%)	1 (2.4%)	–	
Anterior floor of the mouth	10 (17%)	7 (17%)	3 (18%)	
Palatoglossal arch	3 (5.1%)	3 (7.1%)	–	
Ulcerated tumor	27 (46%)	18 (43%)	9 (53%)	0.632
Necrotic tumor	21 (36%)	14 (33%)	7 (41%)	0.645
Chemotherapy	34 (58%)	27 (64%)	7 (41%)	0.152
Radiotherapy	38 (64%)	27 (64%)	7 (41%)	0.913
Recurrence	13 (22%)	7 (17%)	6 (35%)	0.298
Deceased	15 (25%)	4 (9.5%)	11 (65%)	<0.001

Counts (percentage out of the total number from each column) are given, with either chi-square statistical test or Fisher’s exact, unless specified otherwise. ^#^ Median (IQR), Wilcoxon rank-sum statistical test.

**Table 3:** The main histopathological descriptive parameters of oral squamous cell carcinomas.

Variable	All Patients (*N* = 59)	Immunotype A (*N* = 42)	Immunotype B (*N* = 17)	*p*-Value
Histological type				0.321
Conventional	40 (68%)	29 (69.1%)	11 (64.8%)	
Basaloid	9 (15%)	8 (19%)	1 (5.9%)	
Acantholytic	5 (8.5%)	3 (7.1%)	2 (11.7%)	
Papillary	3 (5.1%)	1 (2.4%)	2 (11.7%)	
Spindle cell	2 (3.4%)	1 (2.4%)	1 (5.9%)	
Histologic grade				0.913
G1	6 (10%)	5 (12%)	1 (5.9%)	
G2	45 (76%)	31 (74%)	14 (82.4%)	
G3	8 (14%)	6 (14%)	2 (11.7%)	
Keratinized	20 (34%)	15 (36%)	5 (29%)	0.851
Pathologic T stage				<0.001 **
T1	22 (37.3%)	21 (50%)	1 (5.9%)	
T2	25 (42%)	16 (38%)	9 (52.9%)	
T3	11 (19%)	5 (12%)	6 (35.3%)	
T4a	1 (1.7%)	0	1 (5.9%)	
Pathologic N stage				0.027 *
N0	28 (47%)	23 (54.3%)	5 (29%)	
N1	6 (10%)	3 (7.1%)	3 (18%)	
N2a	3 (5.1%)	2 (4.8%)	1 (5.9%)	
N2b	5 (8.5%)	1 (2.4%)	4 (23.2%)	
N2c	1 (1.7%)	1 (2.4%)	–	
N3b	1 (1.7%)	–	1 (5.9%)	
Nx	15 (25%)	12 (29%)	3 (18%)	
Perineural invasion				0.991
Pn0	35 (59%)	25 (60%)	10 (59%)	
Pn1	24 (41%)	17 (40%)	7 (41%)	
Lymphovascular invasion				
VL0	39 (66%)	30 (71%)	9 (53%)	
VL1	20 (34%)	12 (29%)	8 (47%)	
Depth of invasion				<0.001 **
<5mm	19 (32%)	19 (45.2%)	–	
5–10 mm	30 (51%)	19 (45.2%)	11 (65%)	
>10 mm	10 (17%)	4 (9.6%)	6 (35%)	
^1^ WPI—present	22 (37%)	11 (26%)	11 (65%)	0.008 *

Counts (percentage out of the total number from each column) are given, with either chi-square statistical test or Fisher’s exact, unless specified otherwise. *, **—statistically significant with *p* < 0.05 and *p* < 0.01, respectively. ^1^ WPI (worst pattern of invasion).

## Data Availability

The data that support the fundings on this study are available from the corresponding author upon reasonable request. The data are not publicly available because personal and medical information is strictly protected under the General Data Protection Regulation.

## Abbreviations

The following abbreviations are used in this manuscript:

OSCC	Oral Squamous Cell Carcinoma
NK	Natural Killer
TME	Tumor Microenvironment
TIME	Tumor Immune Microenvironment
HPV	Human Papillomavirus
DOI	Depth of Invasion
WPOI-5	Worst Pattern of Invasion
G1	Well Differentiated
G2	Moderately Differentiated
G3	Poorly Differentiated
TNM	Tumor, Node, Metastasis
p16	Inhibitor of Cyclin-Dependent Kinase 4a
CD3	Cluster of Differentiation 3
CD4	Cluster of Differentiation 4
CD8	Cluster of Differentiation 8
CD20	Cluster of Differentiation 20
RTU	Ready-to-use
CD117	Cluster of Differentiation 117
CD1a	Cluster of Differentiation 1a
CD68	Cluster of Differentiation 68
CD56	Cluster of Differentiation 56
IQR	Interquartile range
OS	Overall Survival
HRs	Hazard Ratios
CIs	Confidence Intervals
T	Pathologic T stage
N	Pathologic N stage
Pn	Perineural Invasion
VL	Lymphovascular Invasion
WPI	Worst Pattern of Invasion
TAMs	Tumor-Associated Macrophages
